# Effectiveness of maternal referral system in a rural setting: a case study from Rufiji district, Tanzania

**DOI:** 10.1186/1472-6963-10-326

**Published:** 2010-12-03

**Authors:** Andrea B Pembe, Anders Carlstedt, David P Urassa, Gunilla Lindmark, Lennarth Nyström, Elisabeth Darj

**Affiliations:** 1 Department of Obstetrics and Gynaecology, Muhimbili University of Health and Allied Sciences, Dar es Salaam, Tanzania; 2 Department of Women's and Children's Health, International Maternal and Child Health (IMCH), Uppsala University, Sweden; 3 Department of Surgery, Central Hospital, Karlstad, Sweden; 4 Department of Community Health, Muhimbili University of Health and Allied Sciences, Dar es Salaam, Tanzania; 5 Department of Public Health and Clinical Medicine, Umeå University, Umeå, Sweden

## Abstract

**Background:**

The functional referral system is important in backing-up antenatal, labour and delivery, and postnatal services in the primary level of care facilities. The aim of this study was to evaluate the effectiveness of the maternal referral system through determining proportion of women reaching the hospitals after referral advice, appropriateness of the referral indications, reasons for non-compliance and to find out if compliance to referrals makes a difference in the perinatal outcome.

**Methods:**

A follow-up study was conducted in Rufiji rural district in Tanzania. A total of 1538 women referred from 18 primary level of care facilities during a 13 months period were registered and then identified at hospitals. Those not reaching the hospitals were traced and interviewed.

**Results:**

Out of 1538 women referred 70% were referred for demographic risks, 12% for obstetric historical risks, 12% for prenatal complications and 5.5% for natal and immediate postnatal complications. Five or more pregnancies as well as age <20 years were the most common referral indications. The compliance rate was 37% for women referred due to demographic risks and more than 50% among women referred in the other groups. Among women who did not comply with referral advice, almost half of them mentioned financial constraints as the major factor. Lack of compliance with the referral did not significantly increase the risk for a perinatal death.

**Conclusion:**

Majority of the maternal referrals were due to demographic risks, where few women complied. To improve compliance to maternal referrals there is need to review the referral indications and strengthen counseling on birth preparedness and complication readiness.

## Background

The WHO estimates that in 2005, 536 000 maternal deaths occurred due to complication of pregnancy and childbirth and more than half of these occurred in sub-Saharan Africa [1]. Furthermore 3.7 million deaths of newborns occur in the first 28 days of life, 50% of these deaths occur in the first 24 hours of life and 75% in the first week of life. There are 32 stillbirths per 1000 deliveries of which 24-37% are intrapartum deaths [2,3]. In the last decade the approach to reduce maternal and perinatal mortality has shifted from the risk approach involving identification of high risk pregnancies which can develop complications to provision of skilled care during delivery and emergency obstetric care when a complication occurs. The shift has been due to the fact that most maternal deaths occur during labour, delivery and the first day postpartum. The complications leading to these deaths are unpredictable but can be treated if diagnosed early [4,5]. The extent to which this approach will reduce maternal and neonatal mortality is not known as the capacity at the primary level of care to deal with complications is limited by the availability of skilled human resources and facilities. Moreover, accessibility to the hospitals during obstetric emergencies remains difficult in most low-resource countries.

Tanzania is a low-resource country which has pyramidal shaped health care infrastructure with dispensaries and health centres as primary level of care facilities at the base and referral hospitals at the apex. The district hospitals are the first referral hospitals for maternal care where most obstetric intervention including surgery and blood transfusion are available. There is high utilization of antenatal care services with more than 94% of pregnant women attending at least once but on the other hand only 47% of deliveries take place in health facilities [6]. Based on the demographic and health survey in 2005 the maternal mortality ratio (MMR) was estimated at 578 per 100,000 live births [6], however, the United Nations arrived at a much higher estimate (950) based on adjustments made on the data collection methods [1].

The Reproductive and Child Health Card 4 (RCHC-4) of the Ministry of Health and Social Welfare (MoHSW) of Tanzania has guidelines for referrals of pregnant women to the hospital. These guidelines do not make a difference between historical risk factors and actual complications in the present pregnancy. Studies in Tanzania and elsewhere have shown that compliancy to referral advice is low [7-10]. For women to comply with referral they have to understand that something is wrong with the pregnancy. Other factors which may hinder acceptance of referral advice include lack of reliable transport, costs involved and perceived quality of care at the hospitals [7,11,12].

The aim of the study was to evaluate the effectiveness of the maternal referral system in a rural district. The evaluation was performed through determining the proportion of women reaching the hospitals after being referred, appropriateness of the referral indications, reasons for non-compliance to the referral and to find out if compliance to referrals makes a difference in the perinatal outcome.

## Methods

### Study area

According to the projection from the 2002 population and housing census the population in Rufiji district in 2007 was estimated at 240,000 with a yearly growth rate of 2.5% [13]. Geographically the Rufiji river intersects the district from West to East dividing it into flood plain, coastal-delta, and plateau zones. The district experiences a heavy rainy season from February to May and a less intense one from October to December. The majority of the population are peasants growing cassava, rice, sorghum and maize as food crops. Cashew nuts and coconuts are the main cash crops available. Commonly their farms are located some distance from the family home and residents shift to temporary dwellings at the farms during the heavy rain season. Transport in the district includes canoes, boats, motor vehicles and bicycles. Most of the roads in the district are unpaved and difficult to pass especially during the rainy season.

The district has two hospitals; the government owned district hospital located in the district town Utete south of the river and the non profit mission hospital Mchukwi located in the northern part of the river. The hospitals serve as a first line centre for women living close by and first referral for all primary level of care facilities. Caesarean section and blood transfusion can only be provided at the hospitals, while vacuum extraction rarely can be provided at primary level of care or hospitals. There are 56 primary level of care facilities of which four are rural health centres (RHCs) owned by the government, 47 government dispensaries and five private dispensaries.

The primary levels of care facilities are the main access point for maternal and child health services in rural areas. They are staffed primarily by clinical officers who are prescribers and most common the in-charge of the facilities, nurses and/or maternal and child health (MCH) aides as trained maternal service providers. In some dispensaries nurse auxiliaries with one year training or on job training on provision of maternal services are providing maternal care. The rural health centers are larger than the dispensaries and have in addition beds for admitting patients. There is no user fee for antenatal and delivery care services except at Mchukwi mission hospital where investigations and operative procedures are charged. Women book for antenatal care at the health facility nearest to them. All the primary level of care facilities provide antenatal and delivery care for low risk women and are supposed to refer women according to referral indications stipulated in the Ministry of Health and Social Welfare RCHC-4.

Only the district hospital has an ambulance which serves all the primary level of care facilities with emergency referrals to the district hospital. Another vehicle owned by the Mchukwi hospital shuttle three times per day between the hospital and the nearby towns. In emergency referrals health workers assist the woman and family to arrange for transport by calling for the ambulance from the district hospital, but they have to pay for fuel.

The RCHC-4 is divided into three sections; the pregnancy care, delivery and immediately after delivery care, and follow up after delivery care. In the first part, the prenatal care, there are three categories of referral indications named A, B and C. A woman with one of the indications in category A should be referred for further investigations while one with indication in category B, should be referred for delivery. A woman requiring referral in category C is supposed to be referred immediately to hospital. The referral indications in category A, B and C are presented in the table below (Table 1).

**Table 1:** Referral indications during pregnancy care according to RCHC-4, Tanzania

*Category A*
Age below 20 years
Ten or more years since last pregnancy
Previous caesarean section^a^
Previous stillbirth/perinatal death (within one week)
Three or more consecutive abortions
Intercurrent illnesses (heart disease, diabetes mellitus, tuberculosis)
*Category B*
≥5 pregnancies
Height <150 cm
Pelvic deformity
First pregnancy at 35 or more years
Previous caesarean section^a ^or vacuum delivery
Postpartum haemorrhage in previous delivery
Retained placenta in the previous delivery
*Category C*
Blood pressure ≥140/90 mmHg
Haemoglobin less than 60% (8.5 gm/dl)
Albumin in urine
Sugar in urine
Gestational age more than 40 weeks
Intrauterine foetal death
Abnormal lie after 36 weeks
Oedema of the legs, face and hands
Suspected twin pregnancy
Fundal height too big or too small for gestation age
Danger signs^b^

^a^Caesarean section is in category A and B.

^b^Danger signs are not stated

In the natal and immediate postnatal period women with referral indication should as well be referred immediately to the hospital. Indications during this period include spontaneous rupture of membranes without labour, labour before 34 weeks, labour for >12 hours/obstructed, abnormal lie or presentation of the baby, vaginal bleeding, variability of foetal heart beats ( < 120 or >160 beats per minute), elevated body temperature of >38 Centigrade, eclampsia or blood pressure ≥140/90 mmHg, haemoglobin <60% (8.5 gm/dl), small pelvis or big baby, meconeum, retained placenta, severe perineal tear and blood loss ≥500 mls.

During the first visit women are screened for referral indications in category A and B. For those identified as in need of referral a tick is made on a specific box on the antenatal card and the woman is informed of the need to go to hospital for further assessment or for delivery. During the subsequent antenatal care visits women given referral advice should be emphasized on the referral advice given. Those referred for delivery are advised to stay near the hospitals at the late months of pregnancy despite there are no maternity waiting homes near the hospitals. Women and their families have to arrange for a place to stay either in guesthouses or by their relatives. If a woman develops any of the indications for referral in category C during her prenatal care, she is referred immediately to hospital. Some women given a referral to hospital for further assessment or delivery actually come back to the primary level of care in labour for delivery. It is up to the discretion of the health worker to decide to re-refer the women or conduct the delivery.

### Sample size and data collection

According to Kielmann et al 1995 and UNICEF 1997, a random sample of 25% to 30% of the health facilities in a district of an average size is usually adequate and feasible to represent a district health service situation [14,15]. All four RHCs and 14 randomly selected dispensaries in the flood plains and plateau zones among those with five or more deliveries per month were included in the study. The delta zone was not included due to difficulties in accessing the area. The primary levels of care facilities included covered 54% of the population in the district.

The sample size of referred women was calculated using the soft ware Epi Info 6. Based on a study in Gutu, Zimbabwe [16] with an antenatal and delivery referral rate of 36%, a desired precision of 5%, 95% confidence interval, and a power of 90% a sample size of 364 referred women could have been sufficient. However to be able to compare with other studies on the use of obstetric care and captured variation of maternal referrals during rain and dry seasons, all maternal referrals to the hospitals from 1 June 2007 to 30 June 2008 were recorded.

A parallel data collection system was established since the routine data collection indicated the risks but no information whether the women were referred to hospital or not. Health workers at the primary level of care facilities received refresh training on the RCHC-4 with an emphasis on the referral indications. Accurate recording of all women referred to hospital during pregnancy, delivery and after delivery was emphasized. During the training, it was emphasized that all health workers should stick to the national guidelines on referral indications and the health workers should repeat advising the women on referral in the subsequent visits. Information on women's socio-demographic characteristics and indications for referral were collected. If a woman had more than one indication for referral, the one associated with worse outcome or needing urgent attention based on obstetrician assessment was taken. A research identification number tag was stapled on the woman's antenatal card. Women that were referred were identified by the trained health workers at the hospitals, who recorded the treatments and outcome of deliveries if delivery takes place at the hospital. The data collection forms were reviewed by the first author in each primary level of care and the health workers completed missing information.

The woman was regarded complied with referral advice when she reached the hospital after being referred from the primary level of care facility due to any of the indications. Women who did not reach the hospitals were identified by comparing the register books in the primary level of care and the hospitals. These women were traced by the primary level of care providers in their respective catchment's areas. Those women contacted were asked about reasons for not going to the hospital and their pregnancy outcome. If the mother had deceased, a relative or anybody who was with the mother during the incident was interviewed.

The structural quality of the hospitals was assessed. Information on qualified staff for provision of emergency obstetric care, functioning operative theatre, blood transfusion facilities, evacuation facilities, functioning vacuum extractor and availability of antibiotics, oxytocics and anticonvulsants.

### Statistical analysis

The referral indications in category A and B in the RCHC-4 card is a mixture of demographic and obstetrical risks, we re-grouped the referral indications in these two categories to make the group containing only the demographic risks and a second group with risks from the obstetrical history and risks related to delivery. The final groups were the demographic risk factors, obstetric historical risks, prenatal complications, natal complications and immediately postnatal complications. Factors which may have higher risks of perinatal mortality were calculated. All factors in the demographic risk and obstetric historical risks groups were used in the calculation of risk of perinatal death. The prenatal and natal complications groups were calculated together and the risk factors included were haemoglobin <60%, blood pressure ≥140/90 mmHg, abnormal lie/presentation, vaginal bleeding, large fundal height for gestation age, labour for >12 hours/obstructed and eclampsia. The risk of perinatal death was not calculated in the postnatal complications group as complications in this group do not affect perinatal outcome. The software SPSS was used for statistical analysis. The risk for perinatal death for women not complying with the referral in relation to those complying was calculated using odds ratio (OR) and 95% confidence intervals (CI).

### Ethical consideration

The protocol was reviewed and approved by the Muhimbili University of Health and Allied Sciences, the Senate research and Publication committee as part of the on going studies on quality assessment and monitoring of maternal referrals in the district. Permission to conduct the study was obtained from the Rufiji District Medical Officer and District Executive Director's office. All participants agreed voluntarily to participate in the study after being informed of the aim and the consent sought from them.

## Results

A total of 5596 women booked for antenatal care in the selected primary levels of care. There were 1538 (28%) women referred to hospitals and out of these 1079 (70%) were referred due to demographic risks, 186 (12%) due to obstetric historical risks, 189 (12%) with prenatal complication and 84 (5.5%) with natal and immediate postnatal complications.

The median age of referred women was 24 years (Range: 14-48) and the median gravidity was 3 (Range: 1-15). A majority of women were married/cohabiting (71%), peasants (90%) and had primary education (54%).

Majority (70%) of the women were referred due to demographic risks. Five or more pregnancies (34%) and age <20 years (30%) were the most common indications contributing to 65% of all referrals. In the group of obstetric historical risks, previous caesarean section made up 65% of all referrals in the group. Low haemoglobin (2.7%), elevated blood pressure (2.3%) and abnormal lie/presentation (2.1%) were the most common indications contributing to 58% of all referrals in the prenatal complications group. Labour for >12 hours/obstructed contributed to 4.2% of all women referred from the primary level of care facilities and 78% of women referred in the group with natal complications.

Reaching the hospital after being given referral advice from the primary level of care facility was regarded as compliance. Out of 1538 women referred 45% complied. The compliance rate for women referred due to demographic risks, obstetric historical risks, prenatal complications and natal complication was 37%, 65%, 56% and 78% respectively. In the obstetric historical risks group women with previous caesarean section (69%), height <150 cm (65%) and history of stillbirth/perinatal death (58%) had the highest compliance rate. Other indication with high compliance rate were blood pressure ≥140/90 mmHg, abnormal lie/presentation and uterus too big for gestational age in the prenatal complication group and labour for >12 hours/obstructed in the natal complication group. The lowest compliance rate was found for ≥5 pregnancies, history of severe bleeding after delivery, ≥10 years since last delivery, intercurrent illnesses and age <20 years (Table 2).

**Table 2:** Number of women referred, proportional complied, and proportional traced after not complying with referral advice according to indications.

Indication for referral	Referred	Complied	Did not comply	
			
			Traced	Lost to follow-up
				
	Number	n (%)	n (%)	n (%)
*Demographic risk factors*				
≥5 pregnancies	529	146 (28)	257 (49)	126 (24)
Age <20 years	465	222 (48)	141 (30)	102 (22)
First pregnancy^a^	37	12 (32)	15 (41)	10 (27)
Intercurrent illnesses^b^	15	7 (47)	5 (33)	3 (20)
≥10 years since last delivery	13	5 (38)	5 (38)	3 (23)
History of severe bleeding after delivery	6	2 (33)	3 (50)	1 (17)
≥3 consecutive abortions	5	0	3 (60)	2 (40)
Pregnancy at ≥35 years^a^	2	1 (50)	1 (50)	0 (0)
First pregnancy at ≥35 years	1	1 (100)		
Other indications^c^	6	4 (67)	1 (17)	1 (17)
Total	1079	400 (37)	431 (40)	248 (23)
*Obstetric historical risks*				
Previous caesarean section	115	79 (69)	18 (16)	18 (16)
Height <150 cm	48	31 (65)	12 (25)	5 (10)
Stillbirth/Perinatal death	19	11 (58)	1 (5.3)	7 (37)
Pelvic deformity	4	0	4 (100)	0 (0)
Total	186	121 (65)	35 (19)	30 (16)
*Prenatal complications*				
Haemoglobin < 60% (8.5 gm/dl)	42	18 (43)	13 (31)	11 (26)
Blood pressure ≥140/90 mmHg	35	23 (66)	6 (17)	6 (17)
Abnormal lie/presentation at >36 weeks	33	18 (55)	10 (30)	5 (15)
Vaginal bleeding	27	12 (44)	8 (30)	7 (26)
Uterus too big for date	26	20 (77)	4 (15)	2 (7.7)
Oedema of hands and face	16	9 (56)	3 (19)	4 (25)
Intrauterine foetal death	4	3 (75)	1 (25)	0 (0)
Albumin in urine	2	1 (50)	0 (0)	1 (50)
Pregnancy >40 weeks	2	1 (50)	0 (0)	1 (50)
Threaten abortion	2	1 (50)	1 (50)	0 (0)
Total	189	106 (56)	46 (24)	37 (20)
*Natal complication*				
Labours for >12 hours/obstructed	64	51 (80)	8 (13)	5 (7.8)
Eclampsia	10	7 (70)	1 (10)	2 (20)
Spontaneous rupture of membrane without labour	7	5 (71)	1 (14)	1 (14)
Cord presentation	1	1 (100)		
Total	82	64 (78)	10 (12)	8 (9.8)
*Immediate postnatal complication*				
Blood loss ≥500 ml	1	1 (100)		
Retained placenta	1	1 (100)		
Total	2	2 (110)		

Total	1538	693 (45)	522 (34)	323 (21)

^a^Indications not in the RCHC-4

^b^Heart disease, diabetes mellitus, tuberculosis, HIV, mental illness, epilepsy

^c^Self request, wanted bilateral tubal ligation, severe abdominal pain, pendulous abdomen, abdominal mass, paraumbilical hernia

(n = 1538)

Financial constraint was the major reason for not complying in 53% in demographic risks, 54% in obstetric historical risks, 46% in prenatal complications, and 70% in the natal complications groups. Other reasons were difficulty acquiring transport, a labour that started suddenly, delivering on the way to hospital and lack of accompaniment to the hospital. Out of 431 women traced in the demographic risks group 15 (3.5%) thought it was not necessary to comply with referral advice given (Table 3).

**Table 3:** Reasons for not attending referral hospital according to referral indication (n = 522)

Reason	Group of referral indication								Total
		
	Demographic risks		Obstetric historical risks		Prenatal complications		Natal complications		
					
	n	%	n	%	n	%	n	%	
Financial difficulties	229	53	19	54	21	46	7	70	276
Difficult to get transport	17	3.9	2	5.7	2	4.3	1	10	22
Thought it was not necessary	15	3.5	2	5.7	0	0	0	0	17
Labour started suddenly	14	3.2	2	5.7	1	2.2	0	0	17
Delivered on the way to hospital	10	2.3	1	2.9	4	8.7	0	0	15
No one to accompany her to hospital	12	2.8	0	0	1	2.2	0	0	13
Delayed to go to hospital	12	2.8	0	0	0	0	0	0	12
The spouse was away on safari	5	1.2	0	0	4	8.7	1	10	10
Aborted	4	0.9	0	0	2	4.3	0	0	6
Labour started at night	4	0.9	0	0	0	0	0	0	4
Other^a^	6	1.4	1	2.9	1	2.2	1	10	9
No reason given	103	24	8	23	10	22	0	0	121

Total	431	99.9	35	99.9	46	100.3	10	100	522

^a^Others includes feared to deliver in hospital, spouse died, refused or sick, no one to care her children at home, disabled, or away on travel

Delivery outcome was available for 653 women who complied with the referral advice and 516 who did not comply. Among women who did not comply, there was no difference in sociodemographic and obstetric characteristics between those found on follow-up and those lost to follow-up. A higher proportion of women referred in the demographic risks group and who did not comply with referral advice delivered at home compared to other referral indication groups in which they delivered more in the primary level of care facilities (Table 4).

**Table 4:** Place of delivery among women complied and did not comply with to referral advice (N = 1169).

Referral indication	Total	Complied			Did not comply	
			
		Home/en route	PLCF	Hospital	Home/en route	PLCF
Demographic risks	804	5 (0.62)	23 (2.9)	349 (43)	266 (33)	161 (20)
Historical obstetric risks	152	0 (0)	2 (1.3)	115 (76)	11 (7.2)	24 (16)
Prenatal complications	140	1 (0.71)	4 (2.9)	91 (65)	20 (14)	24 (17)
Natal complications	73	0 (0)	0 (0)	63 (86)	4 (5.5)	6 (8.2)

Total	1169	6 (0.51)	29 (2.5)	618 (53)	301 (26)	215 (18)

Percentages in brackets

PLCF - Primary level of care facility

There were six maternal deaths. Four complied with the referral and they died at the hospital. Among these, three women died of severe pre-eclampsia/eclampsia. The first woman was referred due to previous caesarean section, the second woman had elevated blood pressure and generalized oedema, and the third one had elevated blood pressure, generalized oedema, twin pregnancy and antepartum haemorrhage. The fourth of these women died of severe postpartum haemorrhage, she was referred on the basis of ≥5 pregnancies. Two women did not comply to the referral advice, one was referred due to threaten abortion and she died two weeks later in the village, and another woman was referred due to ≥5 pregnancies but it was not known when and why she died.

Overall there was no elevated risk for perinatal death among women not complying with the referral advice. Though not significant, perinatal death was elevated in the prenatal and natal complications group in women not complying to referral advice (Table 5).

**Table 5:** Risks of perinatal death among women who did not comply with the referral in comparison with women who complied by referral indications.

Compliance to referral	Perinatal death		OR	95% CI
			
	Yes	No		
*Demographic risks*				
Complied	14	386	1	
Did not comply	15	416	1	0.48-2.1
*Obstetric historical risks*				
Complied	3	118		
Did not comply	0	35		
*Prenatal and natal complications^a^*				
Complied	9	137	1	
Did not comply	2	48	1.6	0.34-7.8

^a^3 women were referred of intrauterine foetal deaths. These are excluded in the prenatal and natal complication group.

Odds ratio (OR) with 95% confidence intervals (CI)

During the study period there were 1608 hospital deliveries, 840 in Utete hospital and 768 in Mchukwi hospital. In each hospital there were four doctors (one medical officer and three assistant medical officers) who provided care to women with obstetric complications. There were three nurse midwives at Mchukwi hospital working in the labour ward and two at Utete hospital. There were a number of MCH Aides and nurse assistants working in the labour ward with the nurse midwives. The number of qualified staff was perceived by the workers to be adequate at Mchukwi hospital, while at Utete hospital it was perceived that more nursing staff and doctors were needed. In both hospitals there was sufficient equipment and facilities, including operating theatres, operative instruments, blood transfusion facilities, antibiotics and oxytocics. Mchukwi hospital had magnesium sulphate solution and diazepam, while Utete had diazepam only for management of eclamptic fits. None of the hospitals had a functioning vacuum extractor.

## Discussion

In this study we found that 28% of women who were booked for antenatal care in the primary levels of care facilities were referred to hospitals. More than two thirds of women referred to the hospitals are in the group of demographic risk factors. Compliance to the referral advice in this group of demographic risks was poor compared to the obstetric historical risks, prenatal, natal and postnatal complications groups. Common reasons mentioned for not complying with the referral advice included financial constraints and difficulty acquiring transport. The two hospitals were considerably well equipped to provide comprehensive emergency obstetric care except for the lack of vacuum extractors.

About one quarter of women booked for antenatal care were referred to hospital during pregnancy, delivery or after delivery. This proportion is lower compared to other studies in developing countries which have shown higher proportions of women needing referral [16-19]. The most common referral indications were ≥5 pregnancies and age <20 years and these accounted for two thirds of all the referrals. Similar findings were found from studies in other parts of sub-Saharan Africa [16,19]. The reason for a high prevalence of these risk factors is that most women in sub-Saharan Africa become pregnant early in their life and many have several pregnancies. Most of the demographic and historical referral indications have been shown to poorly predict occurrence of complications and bad delivery outcome [17,19-21]. Moreover, if all complied with the referral advice, they could overburden the hospitals with otherwise normal deliveries that may lead to a misuse of resources and hampering quality of the maternal care provided.

Compliance to referral advice was lower in the demographic risks group (37%) compared to other risk groups which had more than half of women complying. Low level of referral compliance has been reported from Tanzania and other countries [7,16,22]. A previous qualitative study in the same district showed that the community does not often agree with referral advice based on age <20 years and ≥5 pregnancies without previous complication [12]. In our study the low compliance to these referral indications supports the findings in the qualitative study. Despite the fact that many women did not comply only few (3.3%) thought the referral was not necessary.

Another reason for low level of compliance in the demographic risks group might be due to the criteria for identifying women with risks on the first visit: tick the boxes for the risk and advise the women to go for further assessment or a hospital delivery. This information is given at the first visit. It can be questioned if this is the right time but then the woman and her family can start to prepare for potential referral. The importance of referral should however be emphasized in every subsequent visit by the health worker otherwise the potential risk factor can be forgotten or not considered seriously by the mother and her family. The same reminder should be given for the obstetric historical risks group although in this group women's previous bad experience prompt them to take the referral advice seriously and act upon it.

Financial difficulty and transport problems were the main reason for not complying with the the referral advice. This is in accordance with other studies in developing countries where geographical and financial difficulties deter women from going for the referral [8,20,23,24]. We found that many women referred due to prenatal and natal complications were able to mobilize resources and went to hospital. To improve the referral compliance it is necessary to focus on the referral indications which are more predictive of occurrence of the complications and are as well acceptable to women and the community. To increase women's access to hospital, birth preparedness and emergency readiness should be emphasized. This entails that the woman and her family to devise a plan for delivery at the hospital by identifying a mode of transport, choosing a person to make decisions as well as a person to accompany the woman to the hospital, and allocate funds to be used during referral. Community-based loans and insurance schemes can be used to ease the cost the family incur during referral [25-27]. Hoffman et al suggest the use of motorcycle ambulances at health centres in resource-poor countries as a cheap and time-reducing option to increase timely access for emergency obstetric care [28]. Another way of increasing referral acceptance for women with high risk pregnancies is the use of maternity waiting homes close to the hospitals to reduce access barriers [29]. However, a recent Cochrane review has concluded that there is limited evidence of the use of maternity waiting homes in improving maternal and newborn outcomes [30].

We calculated the risk of perinatal death among women not complying with the referral advice despite knowing that the results might be inconclusive based on small sample and perinatal death being a rare event. The results of a non-elevated risk of perinatal death among non-compliant women to referral advice in the prenatal and natal complications group could be explained by the delays in reaching hospitals. Another reason in the demographic risks group may be that women started labour at home and went to hospital bypassing the primary level of care when there were more serious complications. Studies in Africa have shown that bypassing of the primary level of care facilities and going directly to hospitals is common [7,16]. The absence of vacuum extractors in the hospitals is another bottleneck which may hamper reducing perinatal deaths as cases of foetal distress in the second stage of labour can not be delivered promptly.

Few women were referred because of anaemia (2.7%) and elevated blood pressure (2.3%) in spite of the high prevalence of anaemia and severe anaemia in pregnant women (60% and 27% respectively) [31]. This can be due to poor ability of health workers to screen for these conditions leading to underestimation of the problems [31,32]. More than failure to detect the complications, there may be failure of health workers to refer women with identified risks or complications. Majoko et al [16] reported that nurse midwives in Zimbabwe did not refer 59% of women with previous complications recommended for hospital assessment. It was further noted that 52% of women with elevated blood pressure (≥140/90 mmHg) were not referred. Studies in Zaire have shown that health workers are more willing to refer women with prenatal complications but are reluctant to refer women with risk factors as they are not perceived to predict adverse pregnancy outcome [19,22]. Thus there may be many women not referred to hospitals due to failure of health workers to identify the risks or due to altered perceptions visavis how identified risks affect the outcomes.

## Conclusions

The existing maternal referral system in Rufiji rural district is less effective as majority of the women referred from the primary level of care facilities to the hospitals did not comply. Most of the women were referred due to demographic risk factors and hence a call for review of the national maternal referral indications. Moreover, there is need of strengthening birth preparedness and complication readiness to improve compliance. Further qualitative studies employing in-depth interviews concerning non-compliance to maternal referrals could reveal more information on experiences and perceptions of the women and their families on the referrals.

## Competing interests

The authors declare that they have no competing interests.

## Authors' contributions

ABP, AC, DPU, GL, LN and ED participated in the design of the study. ABP, AC, DPU and ED involved in data collection. ABP performed statistical analysis and drafted the first manuscript. All authors read, commented on and approved the final manuscript.

## Pre-publication history

The pre-publication history for this paper can be accessed here:

http://www.biomedcentral.com/1472-6963/10/326/prepub
